# Treatment-seeking behaviour of nasopharyngeal cancer patients in Yogyakarta, Indonesia

**DOI:** 10.11604/pamj.2018.29.98.12817

**Published:** 2018-01-31

**Authors:** Ema Waliyanti, Fatwa Sari Tetra Dewi, Supriyati Supriyati, Renske Fles

**Affiliations:** 1 School of Nursing, Universitas Muhammadiyah Yogyakarta, Yogyakarta, Indonesia; 2 Department of Health Behavior, Environment and Social Medicine, Faculty of medicine, Universitas Gadjah Mada, Yogyakarta, Indonesia; 3 Department of Head and Neck Surgery and Oncology, Netherlands Cancer Institute, Amsterdam, the Netherlands

**Keywords:** behavior, grounded theory, nasopharyngeal cancer, treatment

## Abstract

**Introduction:**

Nasopharyngeal cancer ranks first among head and neck cancer. About 60-95% of nasopharyngeal cancer patients seek for treatment at advanced stage. Attitudes and behavior of cancer patients in choosing healthcare is affected by the level of socio-economic and cultural backgrounds. Surgery and treatment costs are also the reasons patients to late seek treatment. This study aims to explore what contributes the treatment seeking behavior of nasopharyngeal cancer patients. It conducted in the Yogyakarta, Indonesia.

**Methods:**

As many as 20 patients were interviewed using questionnaire. All interviews were done using Opencode 3.6. To ensure the data validity, triangulation approach, peer debriefing and thick description were done.

**Results:**

it showed that there are five factors that affect the patients in seeking treatment: disease perception, medical services perception, medical expenses, external support and assessment of treatment process.

**Conclusion:**

This study may help to design health education programs to raise public awareness of nasopharyngeal cancer.

## Introduction

Cancer is one of the leading causes of death worldwide, of total 7.6 million deaths, 13% due to cancer [**1**]. Nasopharyngeal Cancer (NPC) is a malignant tumor which ranks fourth out of the five malignant tumors in Indonesia after cervical cancer, breast cancer and skin cancer. NPC ranks first among head and neck cancer [2]. NPC incidence worldwide reached 84.441 cases with mortality rate 51.609, wherein morbidity and mortality rate in male is higher than female [3]. The prevalence of nasopharyngeal cancer in Indonesia is quite high at 1.2 cases per 100,000 inhabitants and 12,000 new cases founded per year [2]. NPC causes 5.7% death in Indonesia [4]. Based on previous research, 60%-95% of NPC patients coming for treatment in the advanced stage [5]. In addition, the lack of knowledge of general practitioner on the health center is also one of crucial factors that lead to late diagnosis of NPC [6]. Attitudes and behavior of cancer patients in choosing a health treatment is influenced by the level of socio-economic and cultural backgrounds. Besides, the fear of treatment side effects, surgery and treatment costs is also the reasons for the delay in seeking treatment [5]. Based on the results of preliminary studies in Dr. Sardjito hospital, total NPC patients who come to the ENT clinic during 2006-2012 were 704 people and the trends increased every year, from 87 people in 2010 to 117 people in 2012. Most of NPC patients (78%) came to hospital at advanced stage (III and IV) and made the prognosis worsen. The purpose of this study is to explore treatment-seeking behavior of NPC patients in Yogyakarta.

## Methods

This study is a qualitative study with grounded theory approach. The research was conducted at Dr. Sardjito hospital and the research subjects were patients with NPC and their families. Twenty informants were selected by purposive sampling. Inclusion criteria for study subjects were patients who seek treatment at Dr. Sardjito hospital, suffering from NPC stage III and IV and not get treatment yet, able to communicate and are willing to become informants. While the criteria for patient's family are close families that often accompany patient during treatment, able to communicate and are willing to become informants. The instrument used in this study is a guideline to conduct indepth interview with NPC patients and families that contains several questions to explore the behavior of people in seeking treatment. To assist data collection, a notebook and a recorder were used as tools. The analysis process started with describing recording data, then data transcribing, open coding, axial coding, selective coding, and the final stage is to develop and describe the situation to become a matrix that describing the factors which influence the centre of phenomenon [7]. The data analysis process were done using Opencode software version 3.6. To ensure the validity of the data in this study, triangulation approach, peer debriefing and thick description were done.

## Results

**Figure 1:**
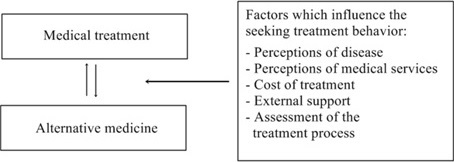
Nasopharyngeal cancer patients seeking treatment behavioral

**Treatment seeking behavior of nasopharyngeal cancer patients in Yogyakart**: Behavior of nasopharyngeal cancer patients in seeking treatment (Figure 1). It shows that patients with nasopharyngeal cancer use both medical and alternative treatments to overcome their illness. These two methods of treatment are used interchangeably. It was happened because of several factors, such as perception of the disease, perception of medical services, medical expenses, external support and assessment of the treatment process. These factors affect the patient behavior in seeking treatment for both medical and alternative medicine.

**Figure 2:**
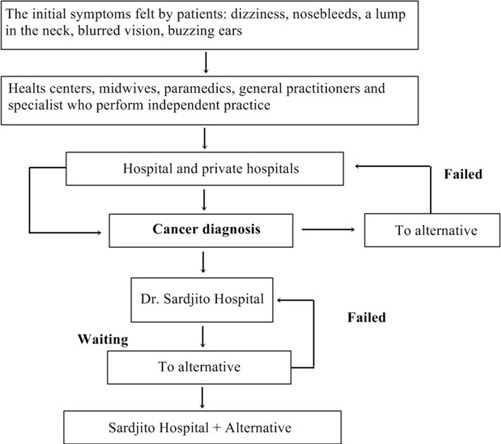
The seeking treatment pattern of nasopharyngeal cancer patients

**The treatment seeking pattern of nasopharyngeal cancer patients**: NPC cancer patients in Yogyakarta use medical and alternative treatments to overcome their illness. The behavior of seeking treatment of NPC patients could be seen in a pattern on the Figure 2.

**Figure 3:**
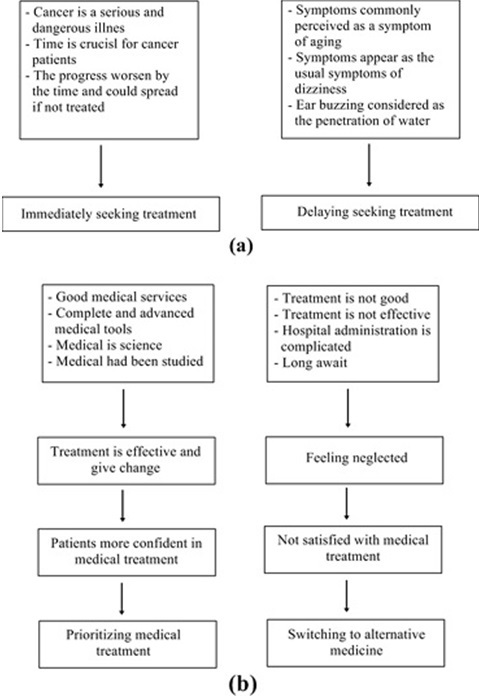
(A) perception of disease; (B) medical services

**Figure 4:**
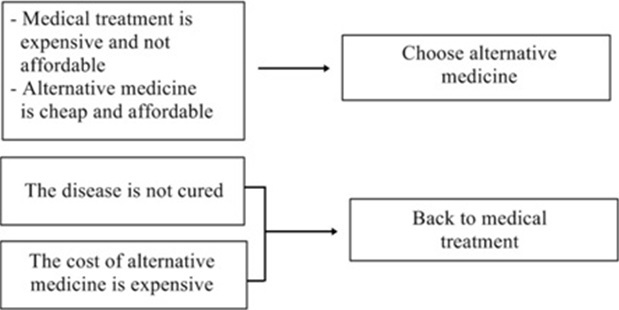
the cost of medical treatment

**Figure 5:**
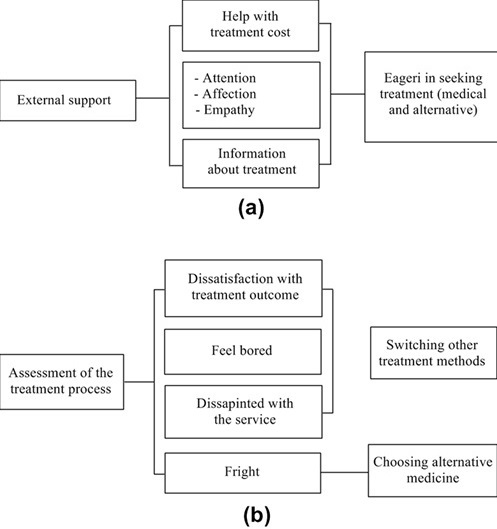
(A) form of external support; (B) assessment of the treatment process

**Factors which influence treatment-seeking behavior**: There are five factors founded that affect patients with NPC in seeking treatment, which are: A) perceptions of disease; how the perception of disease severity could affect health seeking behavior could be seen in the picture; B) perceptions of medical services; how the perception of medical services influence for treatment seeking behavior (Figure 3); C) the cost of treatment; how treatment costs can affect behavior in seeking treatment could be seen in the following (Figure 4); D) the external support in seeking treatment; Figure 5 shows the external support owned by the patients in seeking treatment; E) assessment of the treatment process; how an assessment of the treatment process influence the behavior of the patient in seeking treatment (Figure 5).

## Discussion

Treatment seeking behavior is the behavior of individuals to monitor their body, portray and interpret the symptoms experienced, take action for recovery by using resources to support and involve more formal healthcare system. At first the patient decided to use medical treatment to overcome the disease. While undergoing medical treatment, there are several factors that cause patients and families turn to alternative treatments. Firstly, NPC patients feel disappointed to medical services; the patient and family feel that the service is less satisfactory because the time to get the treatment is too long and patients is not treated immediately, so many patients switch into alternative medicine. Experiences and attitudes to healthcare services is also one of the factors that influence the patient behavior in seeking treatment [8]. Patients who think that the symptoms experienced as a usual aging symptoms, tend to ignore it and delayed the treatment. This caused NPC patients coming late to the health care provider. Health seeking behaviors in patients, starting from the patient's perception of the symptoms. These perceptions based on a merger between the symptoms, cognitive factors and experiences that help someone understand the incidence of disease. Beliefs and one's knowledge of the disease and services available, is very important in influencing an individual to interpret the symptoms and determine the aid to be searched [9]. Instead, people who perceive the illness as a serious one and must be treated, they will immediately seek medical help. This is consistent with Health Belief Model which stated that the perception of disease severity will affect perceptions of threats that affect the behavior of individuals to take immediate action to address the disease [10]. Another factor that affects the patient and family in seeking treatment is the cost of treatment. The cost of medical treatment in Indonesia considered expensive by cancer patients, whereas alternative medicine was considered cheaper, so they chose to go to the alternative treatment. It was also due to the economic level of nasopharyngeal cancer patients in this study, which are poor economic patients so they have to choose more affordable treatment. Based on previous study, patients with lower socio-economic level are more likely to delay health care utilization [11].

## Conclusion

Treatment seeking behavior in patients with NPC in Yogyakarta begin when the first symptoms appears. At first the patient chose to use medical treatment to overcome the disease. While undergoing medical treatment, patients feel the treatment took time too long and not addressed promptly so they turn to alternative treatments. While undergoing alternative treatment most patients founded the treatment less effective and some make the disease becomes more severe, so they return to the medical treatment and some were use alternative medicine as a complementary therapy. The suggestions are, first, for patients and families with NPC need to seeking treatment immediately after the first sign and symptomps occured. Second, health care provider should improve the quality of their services so that the service queue is not take time too long and patient can be treated immediately. The last is health promotion personel need to give health education about NPC to the general public and health professionals so the awareness of this disease will increase.

### What is known about this topic

- Nasopharyngeal Cancer (NPC) is a malignant tumor which ranks fourth out of the five malignant tumors in Indonesia after cervical cancer, breast cancer and skin cancer;
- Attitudes and behavior of cancer patients in choosing a health treatment is influenced by the level of socio-economic and cultural backgrounds.

### What this study adds

- The results showed that there are five factors that affect the patients in seeking treatment for a disease; disease perception, medical services perception, medical expenses, external support and assessment of the treatment process;
- This study may help to design health education programs to raise public awareness of nasopharyngeal cancer.

## Competing interests

The authors declare no competing interests.
